# Metagenomic next-generation sequencing for identifying pathogens in patients with rheumatic diseases and diffuse pulmonary lesions: A retrospective diagnostic study

**DOI:** 10.3389/fcimb.2022.963611

**Published:** 2022-08-31

**Authors:** Juan Jiang, Wei Yang, Yanhao Wu, Wenzhong Peng, Wenjuan Zhang, Pinhua Pan, Chengping Hu, Yisha Li, Yuanyuan Li

**Affiliations:** ^1^Department of Respiratory Medicine, National Key Clinical Specialty, Branch of National Clinical Research Center for Respiratory Disease, Xiangya Hospital, Central South University, Changsha, China; ^2^Center of Respiratory Medicine, Xiangya Hospital, Central South University, Changsha, China; ^3^Clinical Research Center for Respiratory Diseases in Hunan Province, Changsha, China; ^4^Hunan Engineering Research Center for Intelligent Diagnosis and Treatment of Respiratory Disease, Changsha, China; ^5^National Clinical Research Center for Geriatric Disorders, Xiangya Hospital, Changsha, China; ^6^Department of Rheumatology and Immunology, Xiangya Hospital, Central South University, Changsha, China

**Keywords:** metagenomic next-generation sequencing (mNGS), rheumatic diseases, diffuse pulmonary lesions, pulmonary infection, immunosuppressed population

## Abstract

**Objective:**

Lung involvement is a major cause of morbidity and mortality in patients with rheumatic diseases. This study aimed to assess the application value of metagenomic next-generation sequencing (mNGS) for identifying pathogens in patients with rheumatic diseases and diffuse pulmonary lesions.

**Methods:**

This retrospective study included patients who were diagnosed with rheumatic diseases and presenting diffuse pulmonary lesions on chest radiography in Xiangya Hospital from July 2018 to May 2022. Clinical characteristics were summarized, including demographics, symptoms, comorbidities, radiological and laboratory findings, and clinical outcomes. Pulmonary infection features of these patients were analyzed. Furthermore, diagnostic performance of mNGS and conventional methods (including smear microscopy, culture, polymerase chain reaction assay, and serum immunological test) in identifying pulmonary infections and causative pathogens were compared.

**Results:**

A total of 98 patients were included, with a median age of 58.0 years old and a female proportion of 59.2%. Of these patients, 71.4% showed the evidence of pulmonary infections. Combining the results of mNGS and conventional methods, 129 infection events were detected, including 45 bacterial, 40 fungal and 44 viral infection events. Pulmonary mixed infections were observed in 38.8% of patients. The detection rates of mNGS for any pathogen (71.4% vs 40.8%, *P* < 0.001) and mixed pathogens (40.8% vs 12.2%, *P* < 0.001) were higher than that of conventional methods. Moreover, mNGS had a significantly higher sensitivity (97.1% vs. 57.1%, *P* < 0.001) than conventional methods in identifying pulmonary infections, while its specificity (92.9% vs. 96.4%, *P* = 0.553) were comparable to conventional methods. Antimicrobial and antirheumatic treatments were markedly modified based on mNGS results in patients with rheumatic diseases and diffuse pulmonary lesions.

**Conclusions:**

For patients diagnosed with rheumatic diseases and presenting diffuse pulmonary lesions, mNGS is a powerful complement to conventional methods in pathogen identification due to its high efficiency and broad spectrum. Early application of mNGS can provide guidance for precision treatment, and may reduce mortality and avoid antibiotic abuse.

## Introduction

Rheumatic diseases are a group of autoimmune and inflammatory diseases that are characterized by the presence of inflammation and destruction of joints, tissues and internal organs (Apel et al., 2018; Hardy et al., 2020). Lung is a common target organ of autoimmune mediated injury in patients with rheumatic diseases. Depending on the underlying rheumatic diseases, lung involvement associated with rheumatic diseases shows a considerable heterogeneity in incidence, severity, and the components of the involved lung structure (Ha et al., 2018). For patients with rheumatic disease, lung involvement is recognized as a major cause of morbidity and mortality (Luppi et al., 2022). Besides, internal immune dysregulation and secondary immunosuppression caused by antirheumatic therapy make these patients vulnerable to pulmonary infections, including opportunistic infections, which can also present diffuse pulmonary lesions on chest radiography (Di Franco et al., 2017; Wan et al., 2022). Due to the broad range of diagnostic and therapeutic aspects with an increased risk of mortality, rheumatic diseases complicated by diffuse pulmonary lesions are an important and challenging field in the clinical routine of both pulmonologists and rheumatologists.

Early and proper treatments are vital to improve the prognosis of such patients. However, it remains very difficult to distinguish pulmonary infections from non-infectious lung diseases secondary to rheumatic diseases and further identify the causative pathogens. While culture of blood and lower respiratory tract specimen is routinely conducted, its sensitivity is fairly low. Polymerase chain reaction (PCR) assay for specific microbes is widely used for its high sensitivity and specificity. But only one microbe can be detected by one test, thus its application value is limited in mixed infections, which are frequently seen in immunosuppressed patients. More importantly, these conventional methods are far from perfect to detect rare atypical or novel pathogens.

In recent years, mNGS has become increasingly popular in clinical diagnosis of infectious diseases. mNGS is a microbiologic diagnostic tool that detects all nucleic acids of microorganisms whose sequencing data are included in the database library, thus allowing for an unbiased approach to identify pathogens (Simner et al., 2018). Fast reporting, high accuracy, and the ability to detect multiple pathogens by one test make mNGS a promising microbial detection technology (Wilson et al., 2019), especially in complex miscellaneous infectious diseases among immunosuppressed patients (Peng et al., 2021; Nie et al., 2022). Its diagnostic performance has been highlighted in a variety of infectious diseases, such as pneumonia (Xie et al., 2019), blood stream infections (Jing et al., 2021), and central nervous system infections (Ramachandran and Wilson, 2020). However, whether mNGS is advantageous in patients with rheumatic diseases and suspected pneumonia remains unclear. Therefore, we conducted a retrospective study to assess the application value of mNGS in patients with underlying rheumatic diseases and presenting diffuse pulmonary lesions.

## Methods

### Study design and subjects

This retrospective diagnostic study was conducted in Xiangya Hospital, a large-scale tertiary care hospital with more than 3,500 beds. Adults diagnosed with rheumatic diseases and admitted with diffuse pulmonary lesions to the respiratory intensive care unit from July 1, 2018 to May 31, 2022 were consecutively included. Patients were eligible for enrollment if they met all the following criteria: (1) confirmed diagnosis of any kind of rheumatic diseases (Zeng et al., 2008); (2) diffuse pulmonary lesions that newly emerged on chest computed tomography (CT); (3) mNGS test of bronchoalveolar fluid (BALF) was performed. Patients were excluded if they met any of the following criteria: (1) age < 18 years; (2) patients who did not undergo mNGS test; (3) pregnant women; (4) patients with incomplete medical records. Finally, a total of 98 patients were enrolled in this study. The clinical composite diagnosis of pulmonary lesions and causative pathogens for pulmonary infections were determined by two senior experts (LYY and LYS) after discussion with the healthcare team, based on clinical symptoms, laboratory findings, chest radiology, microbiologic tests (including conventional methods and mNGS) and treatment response. This study was approved by the Institutional Review Board and Ethics Committee of Central South University and conducted according to the Declaration of Helsinki. All research data were de-identified and anonymously analyzed.

### Sample processing and DNA extraction for mNGS

BALF samples were processed as described in a previous study of our group (Jiang et al., 2021). Briefly, BALF was mixed with lysozyme and 1 g of 0.5-mm glass beads, and then the mixture was attached to a horizontal platform on a vortex mixer and agitated vigorously at 2800–3200 rpm for 30 min. For nucleic acid extraction, 300 μL of supernatant was transferred to a 1.5-mL centrifuge tube. Subsequently, DNA was extracted using the TIANamp Micro DNA kit (Tiangen Biotech) according to standard procedures.

### DNA library preparation and sequencing for mNGS

The DNA library was constructed by DNA fragmentation, end repair and PCR amplification using MGIEasy Cell-free DNA Library Prep Set (MGI Tech). Agilent 2100 (Agilent Technologies) and Qubit 2.0 (Invitrogen) were used as library quality control. The double-stranded DNA library was converted into single-stranded circular DNA using DNA degradation and circularization. The DNA Nanoballs were generated by rolling circle amplification technology. Qualified DNA Nanoballs were loaded on the chip and then performed 20 M 50-bp single-end sequencing on the MGISEQ-2000 sequencing platform (BGI Genomics) (Jeon et al., 2014).

### Bioinformatic analysis for mNGS

After removing low-quality, short reads (length < 35 base pairs), the human host sequence mapped to the human reference genome using Burrows-Wheeler alignment and low complexity reads (Li and Durbin, 2010; Schmieder and Edwards, 2011), the rest of high-quality data were simultaneously aligned to four large microbial databases, including bacteria (6350 species), fungi (1064 species), viruses (1798 species) and parasites (234 species). The coverage ratio and depth of each microorganism were calculated using BEDTools (Quinlan and Hall, 2010).

Clinically significant microbes detected by mNGS were defined as previously described (Peng et al., 2021). For bacteria (excluding mycobacteria), fungi (excluding molds), viruses, parasites and other atypical pathogens, a microbe detected by mNGS was considered clinically significant when its relative abundance at the species level was more than 30%, and its pulmonary pathogenicity has been recorded in literature. Molds with literature-proven pulmonary pathogenicity were considered as clinically significant when the stringently mapped read number (SMRN) at the species level was more than 10. Oral commensals were not considered as clinically significant microbes regardless of their abundance. Finally, clinically significant microbes were determined as putative pathogens if patients showed suggestive symptoms, laboratory findings and/or radiologic features.

### Clinical data collection

Demographics, symptoms, underlying rheumatic diseases, usage of corticosteroids and immunosuppressants, radiological abnormalities, APACHE II score, laboratory findings, antimicrobial and antirheumatic treatments (Arnold et al., 2021), and clinical outcomes of each patient were obtained through reviewing medical records. Demographics included the age, gender, and smoking history. Laboratory parameters included peripheral white blood cells, neutrophils, lymphocytes, serum procalcitonin, C-reactive protein, serum (1,3)-β-D-glucan, serum galactomannan at admission to the respiratory intensive care unit. A serum (1,3)-β-D-glucan level higher than 95 pg/mL and serum galactomannan S/CO (signal to cut-off ratio) higher than 0.8 were considered as positive according to the manufacturer’s instruction. Clinical outcomes included the use of invasive mechanical ventilation and treatment failure. Treatment failure was defined as the presence of any condition: (1) all-cause death during hospitalization; (2) persistent or worsening symptoms, signs and/or pulmonary lesions on chest imaging at the end of hospitalization. Conventional methods for identifying pathogens included smear microscopy of lower respiratory tract specimen, bacterial and fungal culture of lower respiratory tract specimen and blood, polymerase chain reaction assay of *Epstein-Barr virus* and *Cytomegalovirus*, serum IgM antibody tests for *Mycoplasma pneumoniae*, *Chlamydia pneumoniae*, *Adenovirus*, *Influenza virus*, *Parainfluenza virus*, *Respiratory syncytial virus*, *Coxsackieviruses*, and *Legionella pneumophila*. Mixed infection in lung was defined as two or more kinds of pathogens confirmed in one patient.

### Statistical analysis

Statistical analyses were performed using SPSS 25.0 software (IBM Corp., Armonk, NY, USA). Continuous variables are presented as medians and interquartile ranges, and categorical variables are presented as counts and percentages. Proportions of different infection events were compared using the chi-square or Fisher’s exact tests. The paired chi-square test was used for comparing the pathogen detection rates of mNGS and conventional methods. Kappa test was used for assessing the consistency of mNGS and conventional methods in pathogen identification. Sensitivity, specificity, positive predictive value and negative predictive value were calculated using the clinical composite diagnosis as the reference standard; the chi-square test was used to compare these proportions between mNGS and conventional methods. Significance was fixed at *P* < 0.05.

## Results

### Clinical characteristics of patients

A total of 98 patients diagnosed with rheumatic diseases and diffuse pulmonary lesions were included, and their clinical characteristics were shown in **Table 1**. The median age was 58.0 years. Among these patients, 59.2% were females, and 27.6% were either current or former smokers. The most common clinical manifestations included dyspnea (100%), fever (90.8%), cough (87.8%) and expectoration (71.4%). The top five underlying rheumatic diseases included rheumatoid arthritis (22.4%), polymyositis/dermatomyositis (21.4%), vasculitis (19.4%), systemic lupus erythematosus (14.3%), and Sjögren’s syndrome (10.2%). There were 58 patients using corticosteroids and 22 patients using immunosuppressants before this admission.

**Table 1:** Clinical characteristics of enrolled patients.

Characteristics	Patients (n = 98)
Age (years), median (IQR)	58.0 (49.5-67.0)
Female, n (%)	58 (59.2)
Current or former smoker, n (%)	27 (27.6)
Clinical manifestations Fever, n (%) Cough, n (%) Expectoration, n (%) Dyspnea, n (%)	89 (90.8) 86 (87.8) 70 (71.4) 98 (100)
Rheumatic diseases Rheumatoid arthritis, n (%) Polymyositis/dermatomyositis, n (%) Vasculitis, n (%) Systemic lupus erythematosus, n (%) Sjögren’s syndrome, n (%) Antisynthetase syndrome, n (%) Systemic sclerosis, n (%) IgG4-related disease, n (%) Adult-onset Still’s disease, n (%) Overlap syndrome, n (%)	22 (22.4) 21 (21.4) 19 (19.4) 14 (14.3) 10 (10.2) 6 (6.1) 3 (3.1) 1 (1.0) 1 (1.0) 1 (1.0)
Systemic use of corticosteroids before admission, n (%)	58 (59.2)
Use of immunosuppressants before admission, n (%)	22 (22.4)
Radiological features on chest CT Ground-glass opacity, n (%) Interstitial pattern, n (%) Consolidation, n (%) Nodules, n (%) Pleural effusion, n (%)	72 (73.5) 62 (63.3) 10 (10.2) 17 (17.3) 52 (53.1)
Oxygen index, median (IQR)	128.0 (94.0-171.0)
APACHE II score, median (IQR)	12 (9-15)
Peripheral white blood cells (× 10^9^/L), median (IQR)	9.0 (5.5-11.4)
Peripheral neutrophils (× 10^9^/L), median (IQR)	7.5 (4.4-10.0)
Peripheral lymphocytes (× 10^9^/L), median (IQR)	0.6 (0.4-1.1)
Procalcitonin (ng/mL), median (IQR)	0.36 (0.11-1.62)
C-reactive protein (mg/L), median (IQR)	87.0 (25.0-182.0)
Serum BDG +, n (%)	26/72 (36.1)
Serum galactomannan +, n (%)	8/70 (11.4)
Invasive mechanical ventilation, n (%)	48 (49.0)
Treatment failure, n (%)	23 (23.5)

IQR, interquartile range; CT, computed tomography; APACHE II score, Acute Physiology and Chronic Health Evaluation II score; BDG, (1,3)-β-D-glucan.

On chest CT images, ground-glass opacity (73.5%), interstitial pattern (63.3%), and pleural effusion (53.1%) were frequently seen in these patients. The median oxygen index was 128.0 (94.0-171.0), and the median APACHE II score was 12 (9-15) points. As for laboratory findings, these patients showed significantly lower peripheral lymphocyte counting (median, 0.6× 10^9^/L) and C-reactive protein (median, 87.0 mg/L), while serum procalcitonin level were slightly elevated. Positive rates of serum (1,3)-β-D-glucan and serum galactomannan were 36.1% and 11.4%, respectively. Appropriately half (49.0%) of patients required invasive mechanical ventilation, and 23.5% ended up having treatment failure.

### Pulmonary infection features of patients

**Figure 1:**
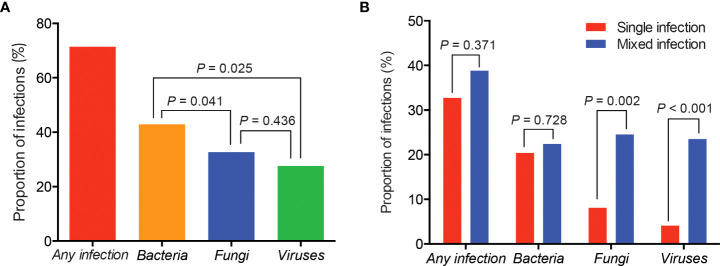
Proportions of different types of infections in patients with rheumatic diseases and diffuse pulmonary lesions. **(A)** Proportions of total, bacterial, fungal and viral infection events; **(B)** Proportions of single and mixed infection events.

**Figure 2:**
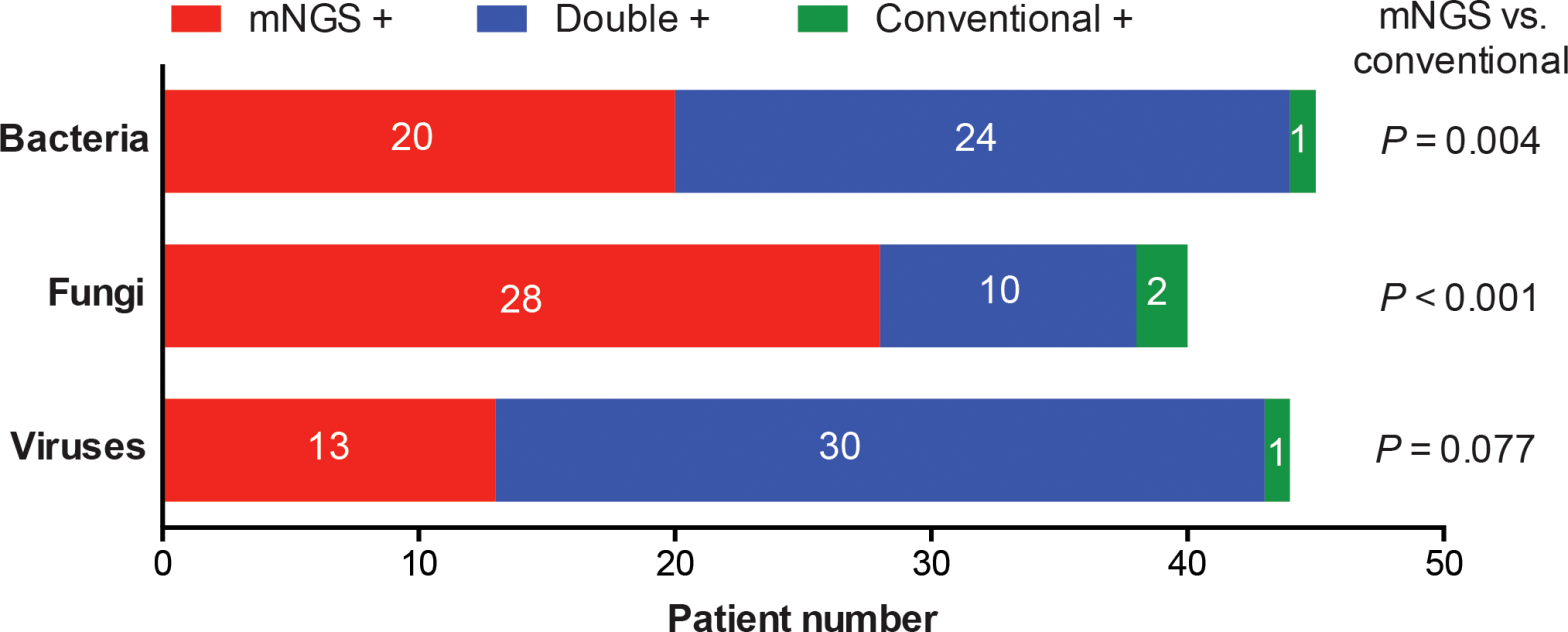
Categorization of infection events detected by mNGS and conventional methods alone or simultaneously. Detection rates of bacteria, fungi or viruses were compared between mNGS and conventional methods.

Of the 98 patients, 71.4% showed the evidence of pulmonary infections, including 42 patients with bacterial infections, 32 with fungal infections and 27 with viral infections (**Figure 1A**). Overall, the proportion of bacterial infections was significantly higher than that of fungal or viral infections (**Figure 1A**). Proportions of mixed infections were 38.8%, 22.4%, 24.5%, and 23.5% for total, bacterial, fungal and viral infections, respectively (**Figure 1B**). In patients infected with fungi or viruses, mixed infection was much more common than single infection (**Figure 1B**). Combining the results of mNGS and conventional methods, 129 infection events were detected, including 45 bacterial infections, 40 fungal infections, and 44 viral infections (**Figure 2**).

**Figure 3:**
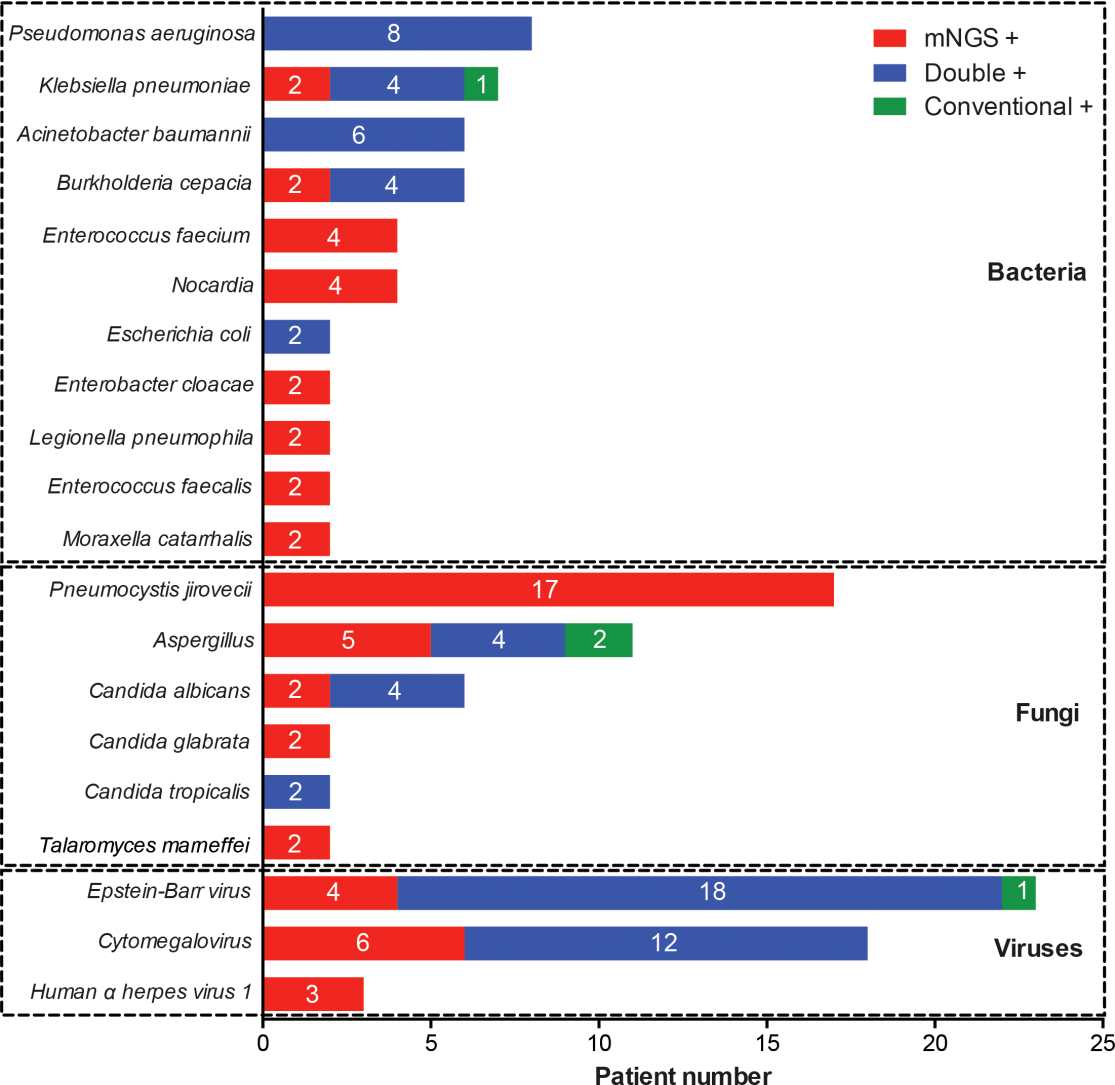
Distribution of pathogenic microorganisms detected by mNGS and conventional methods alone or simultaneously.

The whole pathogen spectrum in patients with rheumatic diseases and diffuse pulmonary lesions was presented in **Figure 3**. *Pseudomonas aeruginosae* was the most commonly detected bacterium (8/98, 8.2%), followed by *Klebsiella pneumonia* (7/98, 7.1%), *Acinetobacter baumannii* (6/98, 6.1%), *Burkholderia cepacia* (6/98, 6.1%), and other bacteria. The most common fungus was *Pneumocystis jirovecii* (17/98, 17.3%), followed by *Aspergillus* (11/98, 11.2%), *Candida albicans* (6/98, 6.1%), and other fungi. *Epstein-Barr virus* (23/98, 23.5%) and *Cytomegalovirus* (18/98, 18.4%) were the most frequently detected viruses. Most of infection events were identified by either mNGS alone or simultaneous mNGS and conventional methods, and only 4 infection events were detected by conventional methods alone.

### Performance of mNGS and conventional methods for identifying pathogens

Results of pathogen number detected by mNGS or conventional methods were shown in **Table 2**. The detection rates of mNGS for identifying any pathogen (71.4% vs 40.8%) and mixed pathogens (40.8% vs 12.2%) were both significantly higher than that of conventional methods, with statistically significant differences (*P* < 0.001). Kappa coefficient (0.344) suggested a mild consistency between mNGS and conventional methods. Diagnostic performance of mNGS and conventional methods in identifying pulmonary infections were further compared. As shown in **Table 3**, mNGS had a significantly higher sensitivity (97.1% vs. 57.1%, *P* < 0.001) and negative predictive value (92.9% vs. 47.4%, *P* < 0.001) than conventional methods, while its specificity (92.9% vs. 96.4%, *P* = 0.553) and positive predictive value (97.1% vs. 97.6%, *P* = 0.896) were comparable to conventional methods.

**Table 2:** Comparison of pathogen species detected by mNGS and conventional methods.

Number of pathogens	mNGS	Conventional methods	*P* value	Kappa coefficient
0	28	58	< 0.001	0.344
1	30	28	0.754	
≥ 2	40	12	< 0.001	

mNGS, metagenomic next-generation sequencing.

**Table 3:** Diagnostic performance of mNGS and conventional methods.

Performance	mNGS	Conventional methods	*P* value
Sensitivity	97.1%	57.1%	<0.001
Specificity	92.9%	96.4%	0.553
Positive predictive value	97.1%	97.6%	0.896
Negative predictive value	92.9%	47.4%	<0.001

mNGS, metagenomic next-generation sequencing.

### Impact of mNGS results on clinical treatments

Modifications on clinical treatments based on mNGS results were summarized in **Table 4**. After the report of mNGS results, 10.2% of patients started systemic use of corticosteroids, 29.6% had increased dosage of corticosteroids, and 8.2% had reduced dosage of corticosteroids. Use of immunosuppressants, therapeutic plasma exchange, and Tocilizumab were initiated in 16.3%, 24.5% and 2.0% of patients, respectively. Antimicrobial treatments were also markedly affected by mNGS results. Among all patients, 62.2% had changed antimicrobial spectrum, 28.6% had narrowed antimicrobial spectrum, and 20.4% had antimicrobial de-escalation. Besides, 28.6% of patients had removed and 13.3% had added at least one antimicrobial agent based on mNGS results.

**Table 4:** Impact of mNGS results on clinical treatments.

Modifications on clinical treatments	Patient number (%)
Systemic use of corticosteroids Any Initiation of corticosteroids Increasing dosage Reducing dosage	47 (48.0) 10 (10.2) 29 (29.6) 8 (8.2)
Initiation of immunosuppressants Initiation of Tocilizumab	16 (16.3) 2 (2.0)
Initiation of TPE	24 (24.5)
Antimicrobial treatments Any Changing antimicrobial spectrum Narrowing antimicrobial spectrum Antimicrobial de-escalation Removing ≥ 1 antimicrobial agent Adding ≥ 1 antimicrobial agent	82 (83.7) 61 (62.2) 28 (28.6) 20 (20.4) 28 (28.6) 13 (13.1)

mNGS, metagemonic next-generation sequencing; TPE, therapeutic plasma exchange.

## Discussion

In this study, we summarized the pulmonary infection features and assessed the diagnostic value of mNGS in patients with rheumatic diseases and diffuse pulmonary lesions. The probability of bacterial, fungal or viral infections is high, and mixed infections are common in these patients. mNGS shows a good ability for detecting pathogens and is advantageous to identifying mixed infections in lung.

Lung involvement in patients with rheumatic disease is common and associated with significant morbidity and mortality (Doyle and Dellaripa, 2017). Meanwhile, the estimated rates of infectious complications can be as high as 26–50% among patients with polymyositis/dermatomyositis or systemic lupus erythematosus (Hsu et al., 2019), and lung is the most frequent infection site (Mok et al., 2011). To distinguish between pulmonary infections and non-infectious diseases is the crucial but challenging step for clinical physicians, even with the efforts of multidisciplinary collaboration. On one hand, conventional microbiological methods have a low yield for pathogen detection. On the other hand, pulmonary lesions mixing infectious with non-infectious diseases are often observed in these patients. Therefore, how to improve the identification of pulmonary infections and causative pathogens is of a great clinical significance for the management of these patients. By combining conventional methods and mNGS, we found that pulmonary infections were confirmed in more than 70% of patients. Mixed infections in lung were observed in 38.8% of patients with rheumatic diseases, in line with a previous study (Chen et al., 2021). Besides, our data showed that mixed infections were more common in patients with fungal or viral infections. These results highlight the importance of fast and comprehensive pathogen identification in patients with rheumatic diseases.

Based on high-throughput sequencing methods, mNGS enables identification of a comprehensive spectrum of potential microbes by a single test, and becomes a powerful approach for the diagnosis of infectious disease. While the usefulness of mNGS in immunocompromised patients with suspected pneumonia has been confirmed by previous studies (Jiang et al., 2021; Peng et al., 2021; Sun et al., 2021), its application value in patients specifically with rheumatic diseases has remained unexplored. We found that the sensitivity of mNGS were markedly higher than that of conventional methods, and the specificity of mNGS was comparable to that of conventional methods. Particularly, mNGS had an advantage in identifying mixed infections over conventional methods, as demonstrated by a higher detection rate (40.8% vs 12.2%) of co-pathogens. Furthermore, mNGS has a unique utility in detecting unculturable and difficult-to-culture microorganisms, such as *Pneumocystis jirovecii* and *Nocardia* (Saubolle and Sussland, 2003; Liu et al., 2018). While PCR test is commonly used for diagnosis of *Pneumocystis jirovecii* pneumonia, simultaneous PCR test of *Pneumocystis jirovecii* was not performed in these patients, due to its inaccessibility in our hospital. Besides, there were a small number of pathogens detected by conventional methods but not by mNGS, including two infection events of *Aspergillus*, one of *Klebsiella pneumonia*, and one of *Epstein-Barr virus*. Although mNGS is well recognized to have a high sensitivity in detecting various pathogens (Parize et al., 2017; Chen et al., 2020), false negative results of mNGS are not rare. Moreover, diagnostic sensitivity of mNGS in fungal infections such as *Aspergillus* is relatively lower (Peng et al., 2021). Difficulty in disrupt the thick polysaccharide cell wall of *Aspergillus* can be an important reason (Zinter et al., 2019). Therefore, the application value of mNGS should be highlighted as a necessary component of the comprehensive pathogen identification system for the diagnosis of pneumonia in patients with rheumatic diseases.

It is worth noting that mNGS results provide important guidance for clinical treatments of patients in this study. As therapeutic strategies for lung diseases secondary to rheumatic diseases are partially contradictory to pulmonary infections, physicians may be overcautious when making treatment plans for patients with rheumatic diseases and diffuse pulmonary lesions. For example, use of immunosuppressive agents can increase the risk of infections and reduce the efficacy of antimicrobial treatment, leading to a clinical dilemma. Without effective interventions, pulmonary lesions usually progress within a short period of time, and consequently cause a high risk of mortality (Atzeni et al., 2018). In this study, both antimicrobial and antirheumatic treatments are greatly modified based on mNGS results. Initial antimicrobial treatments were modified in 83.7% of patients after the report of mNGS results, which could enhance the precision antimicrobial therapeutics and reduce antibiotic side effects. Moreover, corticosteroids, immunosuppressants and therapeutic plasma exchange were not started until the report of mNGS results in a considerable number of patients, indicating that except for detecting possible pathogens, mNGS tests may be helpful in excluding infections in patients with rheumatic diseases. These modifications on clinical treatments may be beneficial to patient prognosis, as suggested by a relatively low rate of treatment failure (23.5%) in this study. However, prospective studies are required to confirm the potential clinical benefits of mNGS in these patients.

**Figure 4:**
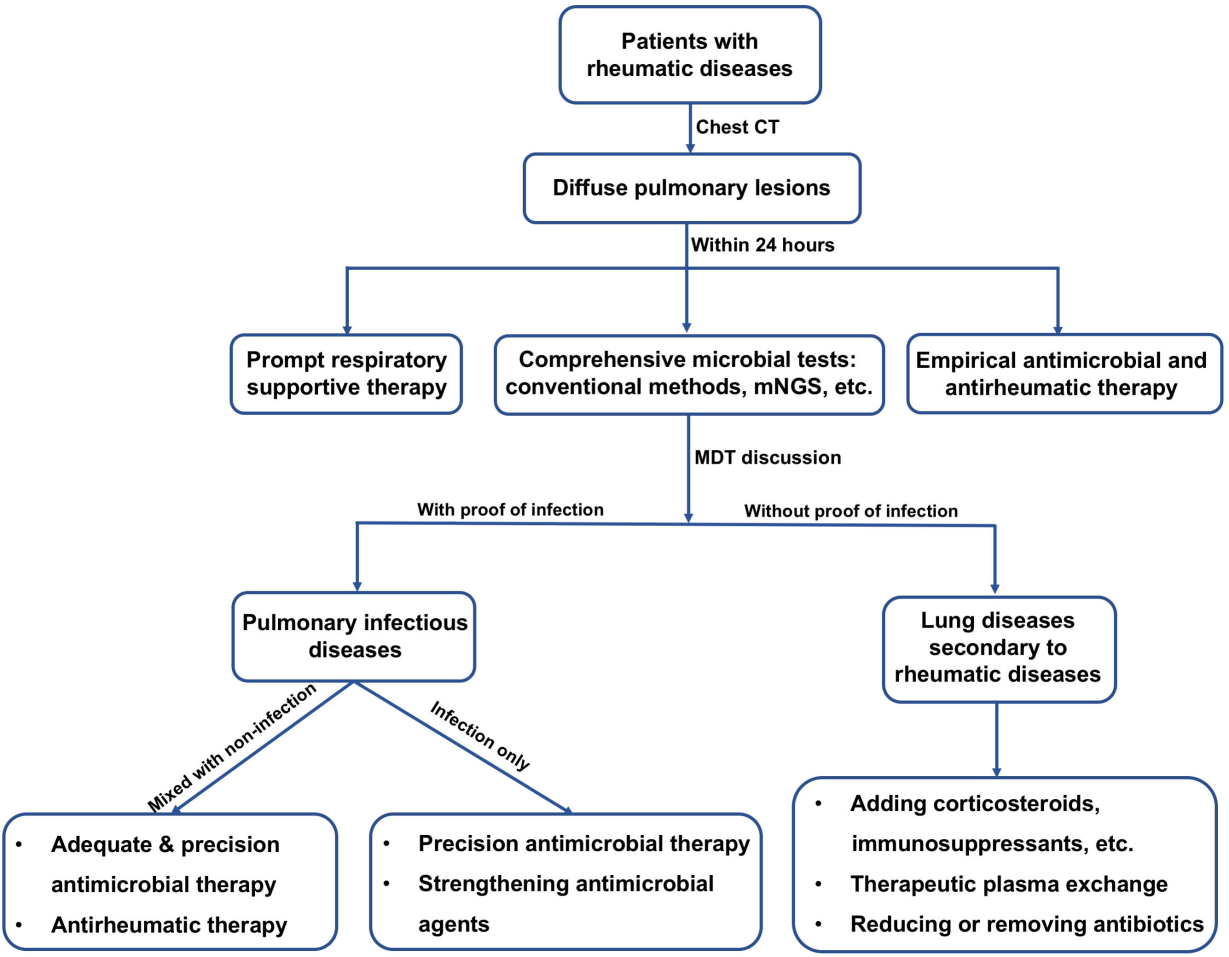
Flow-chart for suggested clinical management of patients diagnosed with rheumatic diseases and presenting diffuse pulmonary lesions. CT, computed tomography; mNGS, metagenomic next-generation sequencing; MDT, multidisciplinary team.

Prompt and appropriate clinical management is directly associated with patient prognosis. Thus, it is crucial to early recognize possible pulmonary infections in patients with rheumatic diseases. This study revealed the infection features in patients with rheumatic diseases and diffuse pulmonary lesions. More importantly, application value of mNGS for identifying pathogens in these patients was highlighted. Therefore, this study has significant clinical implications and provides useful information for physicians. Based on our data and clinical experience, we proposed a guiding strategy flow-chart for clinical management of patients with rheumatic diseases and presenting diffuse pulmonary lesions (**Figure 4**). First of all, we recommend fast and comprehensive microbiological tests including conventional methods and mNGS side-by-side within 24 hours after patient admission. Next, multidisciplinary team discussion should be held to decide the therapeutic strategies of patients. Individual therapeutic strategies must be adopted, avoiding delays in effective treatment and antibiotic abuse.

There are several limitations in the present study. First, this is a single-center retrospective study, thus the intrinsic bias was unavoidable. Second, there is no standard method and widely accepted threshold values for interpreting mNGS results so far, which may affect the objectiveness of mNGS interpretation, especially when distinguishing colonization, infection and contamination. Furthermore, although our data support the diagnostic value of mNGS in patients with rheumatic diseases and diffuse pulmonary lesions, whether its use could improve patient prognosis requires further investigation in large-scale prospective studies.

## Conclusions

In summary, pulmonary infections, including mixed infections, are very common among patients with rheumatic diseases and diffuse pulmonary lesions. The application of mNGS should be highlighted as a powerful complement to conventional methods in clinical management of patients with rheumatic diseases and presenting diffuse pulmonary lesions, due to its high efficiency and broad spectrum of pathogen identification.

## Data availability statement

The original contributions presented in the study are included in the article/supplementary material, further inquiries can be directed to the corresponding authors.

## Ethics statement

The studies involving human participants were reviewed and approved by The Institutional Review Board and Ethics Committee of Central South University. Written informed consent for participation was not required for this study in accordance with the national legislation and the institutional requirements.

## Author contributions

Study concept and design: YYL and YSL. Acquisition of data: JJ, WY, YW, WP, WZ, PP and CH. Statistical analysis: JJ. Analysis and interpretation of data: YYL, YSL and JJ. Drafting of the manuscript: JJ. Critical revision of the manuscript: YYL and YSL. All authors contributed to the article and approved the submitted version.

## Funding

This work is supported by grants from the National Natural Science Foundation of China (82170041 and 82100099), and the Innovative Research Platform of Hunan Development and Reform Commission (2021-212).

## Acknowledgments

We appreciate the professionalism and compassion demonstrated by all the healthcare workers involved in patient care. We also acknowledge all the patients for their involvement in this study.

## Conflict of interest

The authors declare that the research was conducted in the absence of any commercial or financial relationships that could be construed as a potential conflict of interest.

## Publisher’s note

All claims expressed in this article are solely those of the authors and do not necessarily represent those of their affiliated organizations, or those of the publisher, the editors and the reviewers. Any product that may be evaluated in this article, or claim that may be made by its manufacturer, is not guaranteed or endorsed by the publisher.
